# Chloridobis(dimethyl­glyoximato-κ^2^ *N*,*N*′)(ethyl pyridine-4-carboxyl­ate-κ*N*)cobalt(III) chloro­form monosolvate

**DOI:** 10.1107/S1600536812002449

**Published:** 2012-01-25

**Authors:** Ning Wang, Xuzhuo Sun, Dongjin Wan, Jing Chen, Bo Li

**Affiliations:** a Henan University of Technology, School of Chemistry and Chemical Engineering, Zhengzhou 450001, People’s Republic of China

## Abstract

The title compound, [Co(C_4_H_7_N_2_O_2_)_2_Cl(C_8_H_9_NO_2_)]·CHCl_3_, was synthesized as a model complex of vitamin B_12_. The Co^III^ cation displays an approximately octa­hedral coordination environment, being displaced by 0.0240 (15) Å from the mean plane of the four N atoms of the equatorial plane. The O—H distances in the dimethyl­glyoximate hy­droxy groups are 0.89 (6) and 1.14 (6) Å; such long O—H bonds are very common in cobaloxime derivatives. Weak classical O—H⋯N and non-classical C—H⋯Cl hydrogen-bonding interactions further consolidate the crystal packing.

## Related literature

For background on the chemistry of cobaloximes, see: Schrayzer (1968 ▶); Zangrando *et al.* (2003 ▶). For applications of cobaloximes in proton reduction, see: Raza­vet *et al.* (2005 ▶). For related structures, see: Bhuyan *et al.* (2007 ▶); Dutta *et al.* (2009 ▶); Mandal & Gupta (2005 ▶, 2007 ▶); William *et al.* (1973 ▶). For NMR research on O—H⋯O bridges, see: Bakac & Espenson (1984 ▶). For deprotonation of O—H⋯O bridges by BF_3_·Et_2_O, see: Magnuson & Weber (1974 ▶).
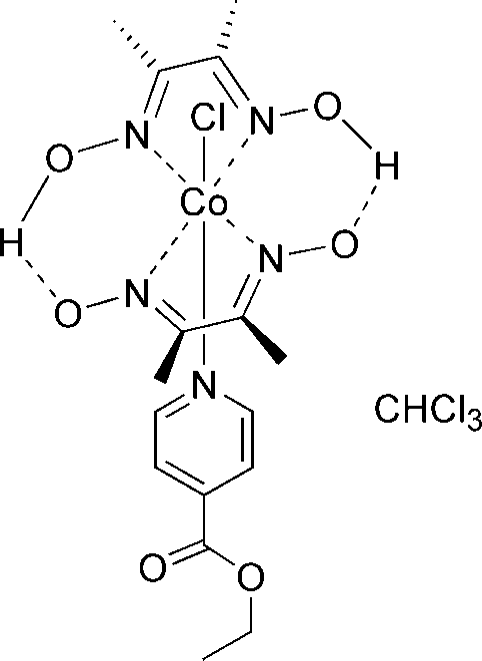


## Experimental

### 

#### Crystal data

[Co(C_4_H_7_N_2_O_2_)_2_Cl(C_8_H_9_NO_2_)]·CHCl_3_
*M* *_r_* = 595.14
Orthorhombic, 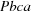

*a* = 10.053 (3) Å
*b* = 22.357 (7) Å
*c* = 23.099 (8) Å
*V* = 5192 (3) Å^3^
*Z* = 8
Mo *K*α radiation
μ = 1.11 mm^−1^
*T* = 293 K
0.29 × 0.14 × 0.06 mm

#### Data collection

Bruker APEXII area-detector diffractometer
Absorption correction: multi-scan (*SADABS*; Bruker, 2007 ▶) *T*_min_ = 0.829, *T*_max_ = 0.935
24393 measured reflections
4558 independent reflections
3483 reflections with *I* > 2σ(*I*)
*R*_int_ = 0.039

#### Refinement

*R*[*F* ^2^ > 2σ(*F* ^2^)] = 0.051
*wR*(*F* ^2^) = 0.158
*S* = 1.08
4558 reflections
310 parameters
H atoms treated by a mixture of independent and constrained refinement
Δρ_max_ = 1.25 e Å^−3^
Δρ_min_ = −0.78 e Å^−3^

### 

Data collection: *APEX2* (Bruker, 2007 ▶); cell refinement: *SAINT-Plus* (Bruker, 2007 ▶); data reduction: *SAINT-Plus*; program(s) used to solve structure: *SHELXS97* (Sheldrick, 2008 ▶); program(s) used to refine structure: *SHELXL97* (Sheldrick, 2008 ▶); molecular graphics: *SHELXTL* (Sheldrick, 2008 ▶); software used to prepare material for publication: *SHELXL97*.

## Supplementary Material

Crystal structure: contains datablock(s) I, global. DOI: 10.1107/S1600536812002449/zl2444sup1.cif

Structure factors: contains datablock(s) I. DOI: 10.1107/S1600536812002449/zl2444Isup2.hkl

Additional supplementary materials:  crystallographic information; 3D view; checkCIF report

## Figures and Tables

**Table 1:** Hydrogen-bond geometry (Å, °)

*D*—H⋯*A*	*D*—H	H⋯*A*	*D*⋯*A*	*D*—H⋯*A*
O2—H2⋯O4	1.14 (6)	1.37 (6)	2.495 (4)	168 (5)
O2—H2⋯N4	1.14 (6)	2.10 (6)	3.004 (4)	133 (4)
O1—H1⋯O3	0.89 (6)	1.60 (6)	2.486 (4)	177 (6)
O1—H1⋯N3	0.89 (6)	2.25 (6)	3.000 (4)	142 (5)
C17—H17*A*⋯Cl1	0.98	2.49	3.437 (6)	163
C6—H6*C*⋯Cl1^i^	0.96	2.79	3.675 (5)	153

Symmetry code: (i) 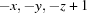.

**Fig. 1.**
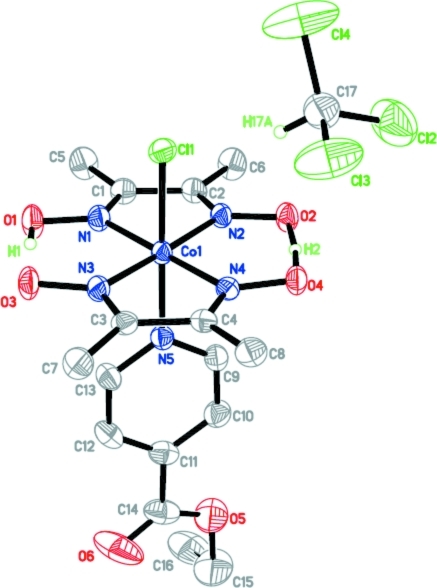
The molecular structure of the title compound. Displacement ellipsoids are drawn at the 30% probability level.

## supplementary crystallographic information

### Comment

The cobaloximes have extensively been used to mimic the vitamin B_12_ coenzyme (Schrayzer, 1968). Recently they also are used to catalyze proton reduction as a functional model of hydrogenase (Razavet *et al.*, 2005). The title compound, [Co(dmgH)_2_(4-(COOEt)C_5_H_4_N)Cl], was prepared as previously described (William *et al.*, 1973). The cobalt atom is coordinated in a distorted-octahedral geometry by four nitrogen atoms of the pseudomacrocyclic [(dmgH)_2_]^2-^ ligand in the equatorial plane and by one chlorine atom and a nitrogen atom of ethyl isonicotinate, respectively, in mutually *trans* positions (N5—Co—Cl1 = 179.33° (10)). The cobalt atom which is linked to four nitrogen atoms belonging to the equatorial plane, displays an approximately octahedral coordination. The mean Co—N bond length is 1.898 Å (3). The mean O—O distance between neighboring dimethylglyoximato oxygen atoms is 2.491 Å (5). These ligands form strong O—H···O bridges with each other which is very common in cobaloxime derivatives. The presence of the O—H···O bridging moieties in cobaloxime derivatives ensures coplanarity of the two ligand molecules and promotes the stability of the cobaloxime molecule (Zangrando *et al.*, 2003). The existence of O—H···O bridging is supported by the NMR data and further substantiated by their chemical behavior with BF_3_.Et_2_O in readily forming an O—BF_2_—O system by deprotonation of the O—H···O bridge (Bakac & Espenson, 1984; Magnuson & Weber, 1974). The distance between O2 and H2 is 1.14 (6) Å indicating a strongly hydrogen bonded nearly symmetric O—H···O system (Bhuyan *et al.*, 2007; Dutta *et al.*, 2009; Mandal & Gupta, 2005, 2007). The Co atom is 0.0240 Å (15) out of the mean plane of the four nitrogen atoms. The plane of the four nitrogen atoms is practically planar.

### Experimental

Co(dmgH)(dmgH_2_)Cl, (3.6 g, 0.01 mol) and ethyl isonicotinate (3.0 g, 0.02 mol) were added to chloroform (90 ml) (William *et al.*,1973). The suspension was stirred for 20 minutes. Then water (30 ml) was added to the flask and the mixture was vigorously stirred for 2 h. The aqueous layer was discarded and the chloroform layer filtered and extracted with water until the washings were nearly colorless. The solution was reduced in volume and the product precipitated by addition of ethanol (95%); yield 69%. Brown single crystals of [Co(dmgH)_2_(4-(COOEt)C_5_H_4_N)Cl] were grown from a CHCl_3_/ethyl acetate solution (v:v = 1:1). ^1^H NMR (400 MHz, CDCl_3_): δ 8.45 (d, 6.4 Hz, 2 H, *o*-H_py_), 7.75 (d, 6 Hz, 2 H, *m*-H_py_), 4.38 (q, 7.2 Hz, 2 H, CH_2_), 2.30 (s, 12 H, N=CCH_3_), 1.35 (t, 7.0 Hz, 3 H, CH_3_).

### Refinement

H1 and H2 were located in difference Fourier maps and their positions and displacement parameters were fully rerfined. All other hydrogen atoms were placed in calculated positions and refined as riding with C—H = 0.93 Å (CH) and 0.97 Å (CH_3_), and with *U*_iso_(H) = 1.5Ueq(C) for the methyl group and *U*_iso_(H) = 1.2U_eq_(C) for all others.

### Crystal data

**Table:**

[Co(C_4_H_7_N_2_O_2_)_2_Cl(C_8_H_9_NO_2_)]·CHCl_3_	*F*(000) = 2432
*M**_r_* = 595.14	*D*_x_ = 1.523 Mg m^−^^3^
Orthorhombic, *P**b**c**a*	Mo *K*α radiation, λ = 0.71073 Å
Hall symbol: -P 2ac 2ab	Cell parameters from 5509 reflections
*a* = 10.053 (3) Å	θ = 2.4–24.8°
*b* = 22.357 (7) Å	µ = 1.11 mm^−^^1^
*c* = 23.099 (8) Å	*T* = 293 K
*V* = 5192 (3) Å^3^	Needle, brown
*Z* = 8	0.29 × 0.14 × 0.06 mm

### Data collection

**Table:**

Bruker APEXII area-detector diffractometer	4558 independent reflections
Radiation source: fine-focus sealed tube	3483 reflections with *I* > 2σ(*I*)
graphite	*R*_int_ = 0.039
phi and ω scans	θ_max_ = 25.0°, θ_min_ = 2.0°
Absorption correction: multi-scan (*SADABS*; Bruker, 2007)	*h* = −11→9
*T*_min_ = 0.829, *T*_max_ = 0.935	*k* = −26→26
24393 measured reflections	*l* = −27→24

### Refinement

**Table:**

Refinement on *F*^2^	Primary atom site location: structure-invariant direct methods
Least-squares matrix: full	Secondary atom site location: difference Fourier map
*R*[*F*^2^ > 2σ(*F*^2^)] = 0.051	Hydrogen site location: inferred from neighbouring sites
*wR*(*F*^2^) = 0.158	H atoms treated by a mixture of independent and constrained refinement
*S* = 1.08	*w* = 1/[σ^2^(*F*_o_^2^) + (0.0892*P*)^2^ + 4.4304*P*] where *P* = (*F*_o_^2^ + 2*F*_c_^2^)/3
4558 reflections	(Δ/σ)_max_ = 0.001
310 parameters	Δρ_max_ = 1.25 e Å^−^^3^
0 restraints	Δρ_min_ = −0.78 e Å^−^^3^

### Special details

**Table:**

Geometry. All e.s.d.'s (except the e.s.d. in the dihedral angle between two l.s. planes) are estimated using the full covariance matrix. The cell e.s.d.'s are taken into account individually in the estimation of e.s.d.'s in distances, angles and torsion angles; correlations between e.s.d.'s in cell parameters are only used when they are defined by crystal symmetry. An approximate (isotropic) treatment of cell e.s.d.'s is used for estimating e.s.d.'s involving l.s. planes.
Refinement. Refinement of *F*^2^ against ALL reflections. The weighted *R*-factor *wR* and goodness of fit *S* are based on *F*^2^, conventional *R*-factors *R* are based on *F*, with *F* set to zero for negative *F*^2^. The threshold expression of *F*^2^ > σ(*F*^2^) is used only for calculating *R*-factors(gt) *etc*. and is not relevant to the choice of reflections for refinement. *R*-factors based on *F*^2^ are statistically about twice as large as those based on *F*, and *R*- factors based on ALL data will be even larger.

### Fractional atomic coordinates and isotropic or equivalent isotropic displacement parameters (Å^2^)

**Table:**

	*x*	*y*	*z*	*U*_iso_*/*U*_eq_	
Co1	0.23591 (5)	0.00646 (2)	0.63484 (2)	0.03477 (19)	
Cl1	0.08443 (9)	0.07824 (5)	0.62035 (4)	0.0473 (3)	
N2	0.2686 (3)	0.00560 (14)	0.55381 (14)	0.0387 (7)	
N4	0.3608 (3)	0.06594 (13)	0.65679 (12)	0.0364 (7)	
O2	0.3577 (3)	0.04186 (12)	0.52883 (11)	0.0489 (7)	
N3	0.1977 (3)	0.00927 (14)	0.71495 (13)	0.0407 (7)	
N5	0.3706 (3)	−0.05592 (13)	0.64728 (13)	0.0406 (7)	
O4	0.4372 (3)	0.09421 (12)	0.61771 (11)	0.0473 (7)	
O3	0.1032 (3)	−0.02516 (14)	0.73944 (11)	0.0558 (7)	
C4	0.3647 (4)	0.07978 (16)	0.71110 (15)	0.0384 (8)	
N1	0.1090 (3)	−0.05257 (15)	0.61321 (13)	0.0443 (8)	
C2	0.2011 (4)	−0.03273 (18)	0.52384 (15)	0.0438 (9)	
O1	0.0261 (3)	−0.07852 (15)	0.65120 (13)	0.0617 (9)	
C3	0.2708 (3)	0.04442 (17)	0.74594 (16)	0.0406 (9)	
C1	0.1055 (4)	−0.06658 (18)	0.55898 (16)	0.0478 (10)	
C6	0.2193 (5)	−0.0395 (2)	0.45984 (18)	0.0661 (13)	
H6A	0.3024	−0.0591	0.4522	0.099*	
H6B	0.2193	−0.0008	0.4420	0.099*	
H6C	0.1478	−0.0630	0.4443	0.099*	
C7	0.2605 (4)	0.0476 (2)	0.81032 (17)	0.0580 (12)	
H7A	0.1723	0.0599	0.8210	0.087*	
H7B	0.3238	0.0760	0.8248	0.087*	
H7C	0.2787	0.0089	0.8266	0.087*	
C9	0.4808 (4)	−0.05867 (19)	0.61441 (18)	0.0514 (10)	
H9A	0.4920	−0.0307	0.5850	0.062*	
C8	0.4506 (4)	0.12779 (18)	0.73592 (18)	0.0522 (10)	
H8A	0.5049	0.1446	0.7059	0.078*	
H8B	0.5065	0.1111	0.7655	0.078*	
H8C	0.3955	0.1585	0.7524	0.078*	
C10	0.5774 (4)	−0.10112 (19)	0.6225 (2)	0.0571 (11)	
H10A	0.6512	−0.1022	0.5982	0.069*	
C13	0.3583 (4)	−0.09637 (18)	0.68994 (17)	0.0518 (10)	
H13A	0.2825	−0.0953	0.7130	0.062*	
C5	0.0100 (5)	−0.1112 (2)	0.5347 (2)	0.0683 (13)	
H5A	−0.0506	−0.1237	0.5645	0.102*	
H5B	0.0582	−0.1452	0.5205	0.102*	
H5C	−0.0389	−0.0933	0.5035	0.102*	
C11	0.5646 (5)	−0.14251 (19)	0.66696 (19)	0.0548 (11)	
C12	0.4530 (5)	−0.1392 (2)	0.70090 (19)	0.0601 (12)	
H12A	0.4414	−0.1659	0.7313	0.072*	
O5	0.7541 (4)	−0.1941 (2)	0.6368 (2)	0.1054 (16)	
O6	0.6596 (6)	−0.2213 (2)	0.7205 (2)	0.134 (2)	
C14	0.6648 (6)	−0.1899 (2)	0.6788 (3)	0.0737 (15)	
C15	0.8560 (7)	−0.2399 (3)	0.6427 (4)	0.130 (3)	
H15A	0.8681	−0.2498	0.6833	0.156*	
H15B	0.9399	−0.2252	0.6277	0.156*	
C16	0.8152 (9)	−0.2930 (4)	0.6107 (3)	0.127 (3)	
H16A	0.8815	−0.3236	0.6149	0.191*	
H16B	0.8053	−0.2831	0.5705	0.191*	
H16C	0.7318	−0.3072	0.6256	0.191*	
Cl2	0.3288 (3)	0.21423 (11)	0.52934 (9)	0.1438 (9)	
Cl3	0.2763 (2)	0.24725 (11)	0.64763 (12)	0.1451 (11)	
Cl4	0.0658 (2)	0.24578 (13)	0.56700 (13)	0.1782 (13)	
C17	0.2161 (6)	0.2156 (3)	0.5858 (2)	0.0785 (15)	
H17A	0.1983	0.1736	0.5954	0.094*	
H2	0.406 (6)	0.064 (3)	0.568 (3)	0.105 (19)*	
H1	0.053 (6)	−0.060 (3)	0.683 (3)	0.10 (2)*	

### Atomic displacement parameters (Å^2^)

**Table:**

	*U*^11^	*U*^22^	*U*^33^	*U*^12^	*U*^13^	*U*^23^
Co1	0.0347 (3)	0.0443 (3)	0.0253 (3)	−0.0059 (2)	0.00124 (18)	0.00086 (19)
Cl1	0.0387 (5)	0.0631 (6)	0.0401 (5)	0.0045 (4)	−0.0007 (4)	0.0016 (4)
N2	0.0389 (17)	0.0465 (18)	0.0306 (17)	−0.0038 (14)	0.0041 (12)	0.0030 (13)
N4	0.0354 (16)	0.0430 (16)	0.0308 (16)	−0.0006 (13)	−0.0008 (12)	0.0025 (13)
O2	0.0527 (17)	0.0618 (17)	0.0321 (14)	−0.0127 (14)	0.0075 (12)	0.0021 (12)
N3	0.0389 (17)	0.0536 (19)	0.0296 (16)	−0.0037 (14)	0.0033 (13)	0.0034 (13)
N5	0.0424 (17)	0.0409 (17)	0.0384 (17)	−0.0040 (14)	−0.0010 (13)	0.0012 (13)
O4	0.0460 (15)	0.0562 (16)	0.0398 (15)	−0.0164 (13)	0.0049 (11)	0.0074 (12)
O3	0.0506 (17)	0.0802 (19)	0.0366 (15)	−0.0215 (15)	0.0116 (12)	0.0071 (14)
C4	0.0384 (19)	0.0416 (19)	0.035 (2)	0.0047 (16)	−0.0036 (15)	0.0002 (15)
N1	0.0430 (18)	0.0542 (19)	0.0358 (18)	−0.0111 (15)	−0.0005 (13)	0.0029 (14)
C2	0.052 (2)	0.051 (2)	0.0293 (18)	−0.0015 (18)	−0.0021 (17)	−0.0057 (17)
O1	0.065 (2)	0.078 (2)	0.0422 (17)	−0.0358 (17)	0.0040 (14)	0.0046 (16)
C3	0.037 (2)	0.056 (2)	0.0287 (18)	0.0056 (17)	−0.0011 (15)	−0.0013 (17)
C1	0.052 (2)	0.055 (2)	0.037 (2)	−0.0067 (19)	−0.0063 (17)	−0.0052 (18)
C6	0.075 (3)	0.090 (4)	0.033 (2)	−0.014 (3)	0.003 (2)	−0.013 (2)
C7	0.057 (3)	0.086 (3)	0.031 (2)	−0.006 (2)	0.0015 (17)	−0.005 (2)
C9	0.047 (2)	0.051 (2)	0.056 (3)	0.0002 (19)	0.0069 (19)	0.0105 (19)
C8	0.056 (2)	0.056 (2)	0.044 (2)	−0.007 (2)	−0.0107 (19)	−0.0058 (18)
C10	0.049 (2)	0.054 (2)	0.068 (3)	0.004 (2)	0.004 (2)	0.002 (2)
C13	0.059 (3)	0.054 (2)	0.042 (2)	−0.001 (2)	0.0035 (18)	0.0085 (19)
C5	0.081 (3)	0.070 (3)	0.053 (3)	−0.029 (3)	−0.013 (2)	−0.007 (2)
C11	0.061 (3)	0.048 (2)	0.055 (3)	0.003 (2)	−0.015 (2)	−0.002 (2)
C12	0.080 (3)	0.052 (2)	0.048 (3)	0.001 (2)	−0.006 (2)	0.012 (2)
O5	0.074 (3)	0.078 (3)	0.164 (5)	0.031 (2)	0.017 (3)	0.036 (3)
O6	0.180 (5)	0.134 (4)	0.089 (3)	0.091 (4)	−0.008 (3)	0.028 (3)
C14	0.084 (4)	0.057 (3)	0.081 (4)	0.016 (3)	−0.028 (3)	0.000 (3)
C15	0.086 (5)	0.098 (5)	0.207 (9)	0.052 (4)	0.016 (5)	0.038 (6)
C16	0.151 (8)	0.114 (6)	0.118 (6)	0.072 (6)	0.003 (5)	0.020 (5)
Cl2	0.159 (2)	0.167 (2)	0.1055 (15)	0.0384 (17)	0.0411 (14)	0.0400 (13)
Cl3	0.1212 (16)	0.178 (2)	0.1355 (19)	0.0474 (15)	−0.0562 (14)	−0.0717 (16)
Cl4	0.1211 (17)	0.229 (3)	0.185 (3)	0.0840 (18)	−0.0778 (17)	−0.087 (2)
C17	0.081 (4)	0.079 (4)	0.075 (4)	−0.007 (3)	−0.009 (3)	0.001 (3)

### Geometric parameters (Å, °)

**Table:**

Co1—N3	1.891 (3)	C7—H7C	0.9600
Co1—N4	1.898 (3)	C9—C10	1.370 (6)
Co1—N2	1.901 (3)	C9—H9A	0.9300
Co1—N1	1.902 (3)	C8—H8A	0.9600
Co1—N5	1.965 (3)	C8—H8B	0.9600
Co1—Cl1	2.2375 (12)	C8—H8C	0.9600
N2—C2	1.294 (5)	C10—C11	1.389 (6)
N2—O2	1.339 (4)	C10—H10A	0.9300
N4—C4	1.293 (5)	C13—C12	1.374 (6)
N4—O4	1.344 (4)	C13—H13A	0.9300
O2—H2	1.14 (6)	C5—H5A	0.9600
N3—C3	1.292 (5)	C5—H5B	0.9600
N3—O3	1.347 (4)	C5—H5C	0.9600
N5—C13	1.343 (5)	C11—C12	1.371 (6)
N5—C9	1.345 (5)	C11—C14	1.487 (6)
O4—H2	1.37 (6)	C12—H12A	0.9300
C4—C3	1.471 (5)	O5—C14	1.325 (7)
C4—C8	1.492 (5)	O5—C15	1.454 (7)
N1—C1	1.292 (5)	O6—C14	1.194 (7)
N1—O1	1.342 (4)	C15—C16	1.456 (11)
C2—C1	1.468 (6)	C15—H15A	0.9700
C2—C6	1.497 (5)	C15—H15B	0.9700
O1—H1	0.89 (6)	C16—H16A	0.9600
C3—C7	1.492 (5)	C16—H16B	0.9600
C1—C5	1.493 (6)	C16—H16C	0.9600
C6—H6A	0.9600	Cl2—C17	1.728 (6)
C6—H6B	0.9600	Cl3—C17	1.705 (6)
C6—H6C	0.9600	Cl4—C17	1.712 (6)
C7—H7A	0.9600	C17—H17A	0.9800
C7—H7B	0.9600		
			
N3—Co1—N4	81.36 (13)	H7A—C7—H7B	109.5
N3—Co1—N2	177.80 (13)	C3—C7—H7C	109.5
N4—Co1—N2	98.96 (13)	H7A—C7—H7C	109.5
N3—Co1—N1	98.27 (13)	H7B—C7—H7C	109.5
N4—Co1—N1	179.30 (14)	N5—C9—C10	122.6 (4)
N2—Co1—N1	81.39 (13)	N5—C9—H9A	118.7
N3—Co1—N5	91.17 (13)	C10—C9—H9A	118.7
N4—Co1—N5	90.15 (13)	C4—C8—H8A	109.5
N2—Co1—N5	91.00 (13)	C4—C8—H8B	109.5
N1—Co1—N5	90.45 (14)	H8A—C8—H8B	109.5
N3—Co1—Cl1	89.10 (10)	C4—C8—H8C	109.5
N4—Co1—Cl1	89.28 (9)	H8A—C8—H8C	109.5
N2—Co1—Cl1	88.73 (10)	H8B—C8—H8C	109.5
N1—Co1—Cl1	90.12 (11)	C9—C10—C11	119.8 (4)
N5—Co1—Cl1	179.33 (10)	C9—C10—H10A	120.1
C2—N2—O2	121.4 (3)	C11—C10—H10A	120.1
C2—N2—Co1	116.3 (3)	N5—C13—C12	122.7 (4)
O2—N2—Co1	122.3 (2)	N5—C13—H13A	118.6
C4—N4—O4	121.5 (3)	C12—C13—H13A	118.6
C4—N4—Co1	116.6 (2)	C1—C5—H5A	109.5
O4—N4—Co1	121.9 (2)	C1—C5—H5B	109.5
N2—O2—H2	102 (3)	H5A—C5—H5B	109.5
C3—N3—O3	121.0 (3)	C1—C5—H5C	109.5
C3—N3—Co1	116.5 (3)	H5A—C5—H5C	109.5
O3—N3—Co1	122.4 (2)	H5B—C5—H5C	109.5
C13—N5—C9	117.3 (4)	C12—C11—C10	117.6 (4)
C13—N5—Co1	121.5 (3)	C12—C11—C14	119.2 (5)
C9—N5—Co1	121.2 (3)	C10—C11—C14	123.2 (5)
N4—O4—H2	102 (2)	C11—C12—C13	120.0 (4)
N4—C4—C3	112.5 (3)	C11—C12—H12A	120.0
N4—C4—C8	124.3 (3)	C13—C12—H12A	120.0
C3—C4—C8	123.2 (3)	C14—O5—C15	117.3 (5)
C1—N1—O1	120.8 (3)	O6—C14—O5	125.3 (5)
C1—N1—Co1	116.2 (3)	O6—C14—C11	122.5 (6)
O1—N1—Co1	123.0 (2)	O5—C14—C11	112.1 (5)
N2—C2—C1	112.9 (3)	O5—C15—C16	109.1 (7)
N2—C2—C6	122.1 (4)	O5—C15—H15A	109.9
C1—C2—C6	125.0 (4)	C16—C15—H15A	109.9
N1—O1—H1	99 (4)	O5—C15—H15B	109.9
N3—C3—C4	112.9 (3)	C16—C15—H15B	109.9
N3—C3—C7	122.8 (4)	H15A—C15—H15B	108.3
C4—C3—C7	124.3 (3)	C15—C16—H16A	109.5
N1—C1—C2	113.1 (3)	C15—C16—H16B	109.5
N1—C1—C5	122.9 (4)	H16A—C16—H16B	109.5
C2—C1—C5	123.9 (4)	C15—C16—H16C	109.5
C2—C6—H6A	109.5	H16A—C16—H16C	109.5
C2—C6—H6B	109.5	H16B—C16—H16C	109.5
H6A—C6—H6B	109.5	Cl3—C17—Cl4	111.2 (3)
C2—C6—H6C	109.5	Cl3—C17—Cl2	114.0 (3)
H6A—C6—H6C	109.5	Cl4—C17—Cl2	113.2 (4)
H6B—C6—H6C	109.5	Cl3—C17—H17A	105.9
C3—C7—H7A	109.5	Cl4—C17—H17A	105.9
C3—C7—H7B	109.5	Cl2—C17—H17A	105.9

### Hydrogen-bond geometry (Å, °)

**Table:**

*D*—H···*A*	*D*—H	H···*A*	*D*···*A*	*D*—H···*A*
O2—H2···O4	1.14 (6)	1.37 (6)	2.495 (4)	168 (5)
O2—H2···N4	1.14 (6)	2.10 (6)	3.004 (4)	133 (4)
O1—H1···O3	0.89 (6)	1.60 (6)	2.486 (4)	177 (6)
O1—H1···N3	0.89 (6)	2.25 (6)	3.000 (4)	142 (5)
C17—H17A···Cl1	0.98	2.49	3.437 (6)	163
C6—H6C···Cl1^i^	0.96	2.79	3.675 (5)	153

Symmetry codes: (i) −*x*, −*y*, −*z*+1.
